# Biodetoxification of toxins generated from lignocellulose pretreatment using a newly isolated fungus, *Amorphotheca resinae *ZN1, and the consequent ethanol fermentation

**DOI:** 10.1186/1754-6834-3-26

**Published:** 2010-11-22

**Authors:** Jian Zhang, Zhinan Zhu, Xiaofeng Wang, Nan Wang, Wei Wang, Jie Bao

**Affiliations:** 1 State Key Laboratory of Bioreactor Engineering, East China University of Science and Technology, 130 Meilong Road, Shanghai 200237, China

## Abstract

**Background:**

Degradation of the toxic compounds generated in the harsh pretreatment of lignocellulose is an inevitable step in reducing the toxin level for conducting practical enzymatic hydrolysis and ethanol fermentation processes. Various detoxification methods have been tried and many negative outcomes were found using these methods, such as the massive freshwater usage and wastewater generation, loss of the fine lignocellulose particles and fermentative sugars and incomplete removal of inhibitors. An alternate method, biodetoxification, which degrades the toxins as part of their normal metabolism, was considered a promising option for the removal of toxins without causing the above problems.

**Results:**

A kerosene fungus strain, *Amorphotheca resinae *ZN1, was isolated from the microbial community growing on the pretreated corn stover material. The degradation of the toxins as well as the lignocelluloses-derived sugars was characterized in different ways, and the results show that *A. resinae *ZN1 utilized each of these toxins and sugars as the sole carbon sources efficiently and grew quickly on the toxins. It was found that the solid-state culture of *A. resinae *ZN1 on various pretreated lignocellulose feedstocks such as corn stover, wheat straw, rice straw, cotton stalk and rape straw degraded all kinds of toxins quickly and efficiently. The consequent simultaneous saccharification and ethanol fermentation was performed at the 30% (wt/wt) solid loading of the detoxified lignocellulosic feedstocks without a sterilization step, and the ethanol titer in the fermentation broth reached above 40 g/L using food crop residues as feedstocks.

**Conclusions:**

The advantages of the present biodetoxification by *A. resinae *ZN1 over the known detoxification methods include zero energy input, zero wastewater generation, complete toxin degradation, processing on solid pretreated material, no need for sterilization and a wide lignocellulose feedstock spectrum. These advantages make it possible for industrial applications with fast and efficient biodetoxification to remove toxins generated during intensive lignocellulose pretreatment.

## Background

Pretreatment is a requisite step in overcoming the biocalcitrance of lignocellulose and remains one of the most expensive steps for cellulosic ethanol production [1]. This harsh step generally involves strong chemical or physicochemical conditions to break the lignocellulose structure and release monosaccharide sugars by cellulase enzymes [2]. Various lignocellulose derived toxins, including furan derivatives (furfural and 5-hydromethylfurfural (HMF)), organic acids (acetic acid, formic acid, and ferulic acid) and lignin derivatives (vanillin, 4-hydroxybenzaldehyde, guaiacol, and phenol), are generated during pretreatment processing [3,4]. These toxins severely inhibit the consequent enzymatic hydrolysis and ethanol fermentation [2,5]. Therefore, a detoxification step to remove the toxins for the consequent hydrolysis and fermentation is unavoidable.

Various detoxification methods had been tried, such as water washing, overliming, vaporization and ion exchange absorption [3,6]. However, these methods resulted in many negative outcomes, including massive freshwater usage and wastewater generation, loss of the fine lignocellulose particles and fermentative sugars and incomplete removal of inhibitors [7]. Among the most frequently used methods, water washing, considerable pretreated lignocellulose solids were lost during the washing and liquid-solid separation step, thus leading to the loss of ethanol of at least the same percentage. Furthermore, the considerable amount of water used led to high cost of the downstream wastewater treatment. Finally, the high water content in the pretreated feedstock led to the low ethanol titer in the consequent fermentation and then the high energy cost in the distillation.

An alternate option for removing toxins without causing these problems is biodetoxification, which relies on microorganisms to degrade the toxins as part of their normal metabolism by secreting peroxidase or laccase enzymes into the hydrolysate [8-11]. Biodetoxification has many advantages, such as no loss of cellulose solids, greatly decreased use of water, and thus high concentrations of solids for fermentation. However, the current biodetoxification method applied only to the liquid hydrolysate system, in which the cellulose was hydrolyzed under toxin inhibition to cellulase enzymes and the toxin concentrations had been diluted; thus the degradation rate was decreased. Furthermore, the reduced sugars in the hydrolysate at high concentrations might be consumed. The slow biodegradation rate of toxins significantly limited its practical applications. No fermentation practice was carried out using the detoxified materials as the feedstock for ethanol production.

In this study, a unique fungal microorganism was isolated from the natural habitat environment on the pretreated lignocellulose material, which grew faster than other microorganisms on the pretreated corn stover material. After several rounds of screening, the biodegradation strain was selected and identified as the *Amorphotheca resinae *fungus, a species of kerosene strains, and then named *A. resinae *ZN1. The inhibitor removal of *A. resinae *ZN1 was tested, and it was proven that *A. resinae *ZN1 adapted to the pretreated lignocelluloses-based environment perfectly, grew quickly using the sole inhibitor as the carbon source and preserved the cellulose component well. Besides, in contrast to the previous methods, the detoxification in this work was carried out by the solid-state fermentation on the pretreated lignocellulose materials, thus directly degrading the toxins at its high concentrations. The detoxification using *A. resinae *ZN1 was applied both on the steam explosion and on dilute acid-pretreated corn stover, and then the detoxified corn stover was used for simultaneous saccharification and ethanol fermentation. The detoxification method was applied to various lignocellulose feedstocks and was found to work perfectly. The detoxification strain and the method used in this work provided an effective detoxification method for the utilization of lignocellulose for bioethanol production with higher industrial application potential than previously used methods.

## Methods

### Raw materials and pretreatment

Corn stover (CS) was grown in Jilin, China, and harvested in fall 2007. Rice straw and cotton stalk were grown in Hubei, China, and harvested in 2008. Wheat straw and rape straw were grown in Henan, China, and harvested in 2008. After collection, the materials were milled coarsely on a beater pulverizer (SF-300; Ketai Milling Equipment, Shanghai, China) and screened through mesh with a circle diameter of 10 mm. The milled raw materials were washed to remove the field dirt, stones and metals; dried; and then stored in sealed plastic bags for use.

The steam explosion pretreatment was performed on CS only. The milled CS materials were steam heated to 210°C, 2.2 MPa, for 4 minutes, then the pressure was released quickly [12]. Only saturated steam was used, and no chemicals were added. The pretreated CS contained approximately 50% dry solid matter (DM) and was stored at 4°C for use.

The dilute sulfuric acid pretreatment was performed on all the materials, including CS, rice straw, wheat straw, rape straw and cotton stalk. The pretreatment reactor was a self-made stainless cylinder with the working volume of 10 L, and 800 g of each feedstock material were filled in each operation. The feedstock was presoaked with diluted sulfuric acid solution with the solid (dry material) to liquid (5.0% (wt/wt) sulfuric acid solution) ratio of 2:1 (wt/wt). The presoaked wet materials were fed into the reactor, and the hot steam was jetted directly into the reactor to 190°C, 1.2 MPa, for 3 minutes. All the sulfuric acid solution and the steam-condensed water were absorbed into the solid material to give a DM content of 50% (wt/wt). No free water was generated during the pretreatment. The pretreated materials were released from the reactor and stored at 4°C. The most frequently used pretreatment method in this work is the dilute sulfuric acid method unless mentioned otherwise.

### Enzymes and ethanol fermentation strain

The cellulase enzyme used was Accellerase 1000 from Genencor International (Rochester, NY, USA). The filter paper activity and the cellobiase activity were determined to be 65.8 FPU/ml and 152.0 IU/ml, respectively. One unit of filter paper cellulase (FPU) was defined as the amount of enzyme which produces 2.0 mg of reducing sugar from 50.0 mg of filter paper within 1 h. The detailed procedures for determination of cellulase and cellobiase activities were described in Zhang *et al*. [13].

A thermo-and inhibitor-tolerant mutant strain *Saccharomyces cerevisiae *DQ1 was used for ethanol fermentation. The culture solution was aliquoted into 1-mL vials containing 30% (wt/wt) glycerol and stored in a -80°C. A vial of *S. cerevisiae *DQ1 was taken from the -80°C freezer and directly inoculated in the seeding culture for the purpose of keeping all the seeding strains the same. The adaptation procedure of the strain *S. cerevisiae *DQ1 was described in detail by Zhang *et al*. [13].

### Isolation of detoxification strains

The steam explosion-pretreated CS samples were exposed to ambient air for 2 weeks and stored at 4°C in a refrigerator for months after being transported from the cold northeast China region to the warm southern Shanghai area. The original detoxification strains were isolated using a three-step screening procedure as described in the next three subsections.

#### Strain isolation

Ten grams of the pretreated CS samples were diluted with 90 ml of sterilized water and incubated for 2 h at 30°C and 180 rpm to obtain the 1 × 10^-1 ^suspension. The suspension was further diluted into 10^-2^, 10^-3 ^and 10^-4 ^suspensions. The 10^-4 ^dilution was streaked onto the potato-dextrose-agar (PDA) plates containing 200 g/L potato extract juice, 20 g/L glucose and 20 g/L agar for enrichment culture. The plates were incubated for 5 days at 25°C, and the colonies were restreaked onto the PDA plates on the basis of the morphology and color of the colonies. Then the single colony was isolated, and each colony was restreaked for five generations to obtain the purified single colony.

#### Initial screening

The screening medium was prepared by adding the toxic compounds of 6.0 g/L acetic acid, 1.0 g/L furfural, and 1.5 g/L HMF into the PDA medium. The isolated colonies were streaked onto the screening medium and incubated for 5 days at 25°C. Then the toxin tolerant strains were selected from the PDA medium containing toxins.

#### Detoxification screening

The selected colonies were further quantitatively screened by their toxin degradation abilities on the steam explosion-pretreated CS samples. Ten grams of the pretreated CS were inoculated with 1 ml of diluted suspension (about 1 × 10^6 ^spores/mL) and incubated in the 250-mL flasks at 25°C for 4 days. A quantity of 44 ml of the citrate acid buffer (100 mM, pH 4.8) and 1.25 ml of Accellerase 1000 (15.0 FPU/g DM) were added to the flasks to reach solid loading of 10% (wt/wt). The enzymatic hydrolysis lasted for 12 h at 50°C and 150 rpm in a water bath shaking incubator. The steam explosion-pretreated CS without strain inoculation was used as the control. The initially selected strains with better inhibitor-degrading performance were selected for further experimentation.

### Molecular identification of the detoxification strain

The selected strains were cultured in the PDA medium, and 8 mg of the dry mycelia were collected. The genomic DNA was extracted using the Qiagen DNeasy Tissue Kit (Qiagen, Valencia, CA, USA) and purified following the "Yeast Genomic DNA Purification Protocol" discussed in the attached handbook. The 18 S rDNA internal transcribed spacer (ITS) sequence was amplified by the universal primers ITS1 (5'-TCCGTAGGTGAACCTGCGG-3') and ITS4 (5'-TCCTCCGCTTAGATATGC-3'). The polymerase chain reaction (PCR) products were purified using the PCR Purification Mini Kit (Omega Biotek, Norcross, GA) and sequenced by Shanghai Biotech Service (Shanghai, China). The ITS sequences were blasted in the National Center for Biotechnology Information database http://www.ncbi.nlm.nih.gov/, and the phylogenetic trees were constructed using Bioedit 7.0 http://www.mbio.ncsu.edu/bioedit/bioedit.html and Mega 4 software http://www.megasoftware.net/ using the neighbor-joining method.

### Growth assay of isolates on pretreated CS material

The growth performance under different pH and temperature conditions was assayed by counting the colony numbers. One milliliter of the selected strain (1 × 10^6 ^spores) suspension was inoculated onto 10 g of the pretreated CS and incubated for 3 days at 25°C. The CS was washed with 90 ml of sterilized water, shaken for 2 hours at 25°C and 180 rpm and then diluted to 10^-3 ^or 10^-4 ^fold. A quantity of 0.1 ml of the suspension was taken, spread onto the PDA plates and incubated for 4 days at 25°C. Then the colony numbers on PDA plates were counted. The growth assay under different oxygen levels was operated using two flasks, one filled with nitrogen and sealed with a rubber stopper and the other covered only with a cotton stopper.

### Growth assays of isolates using toxins as the sole carbon sources

The detoxification performance of the isolates using the inhibitor substances as the sole carbon sources was assayed by observation of the mycelia growth on the pretreated CS solids. The dilute sulfuric acid-pretreated CS was thoroughly washed with deionized water until no inhibitor substances from the washout solution were found on performing high performance liquid chromatography (HPLC). Three grams of such thoroughly washed, dried CS were mixed with various toxins (each toxin was dissolved in 10 ml of sterilized water) and used for the solid-state culture. Two milliliters of the spore suspension (1 × 10^6 ^ml) were inoculated onto the 3 g of the mixed CS in 250-ml flasks and cultured for 9 days at 25°C. The mycelia formation in the flask was observed periodically.

### Detoxification assays of toxins by the isolates

The biodetoxification performance of the isolates was assayed by mixing an extra amount of toxic inhibitor substances onto the pretreated CS and then inoculated with the spore suspension of the isolates. Five grams of the pretreated CS were mixed with each of the inhibitor substances, and then 8 ml of sterilized deionized water was added. Next, 1 ml of the isolate suspension (1 × 10^6^) was inoculated onto the CS. The toxic inhibitors supplemented included acetic acid, formic acid, levulinic acid, furfural and HMF. First, the substances were added separately, and then the toxin mixtures were added. The culture was carried out for 5 days at 25°C, then the cellulase enzyme was added for simultaneous saccharification and ethanol fermentation (SSF) assay at the conditions of 15.0 FPU/g DM, 10% (wt/wt) of the detoxified CS solid loading, pH 4.8 (100 mM citrate buffer), 50°C and 150 rpm in the water bath shaking incubator for 12 h. Then *S. cerevisiae *DQ1 was added at a 10% (vol/vol) inoculation ratio for another 12 h at 37°C. The samples were taken at 12-h intervals for HPLC analysis of toxins, glucose and ethanol.

### SSF at high solids loading

Different lignocellulose materials, including CS, rice straw, wheat straw, rape straw and cotton stalk were pretreated using steam explosion or dilute acid methods and then detoxified at 25°C for 3 days with the selected detoxification strain. The SSF at the solid loading of 30% (wt/wt, dry base) was operated in a 5-L helical stirring bioreactor as described by Zhang *et al*. [13]. In the prehydrolysis stage, Accellerase 1000 was fed into the bioreactor at the dosage of 15.0 FPU/g DM, followed by feeding of the detoxified feedstocks into the bioreactor within 12 h at 50°C and 150 rpm. Then the temperature was reduced to 37°C, and the *S. cerevisiae *DQ1 seeds were inoculated into the hydrolysate at the ratio of 10% (vol/vol). pH was maintained at 5.0 using a 5 M NaOH solution and a 1 M H_2_SO_4 _solution. The SSF operation continued for 60 h, and the samples were withdrawn at regular intervals.

The procedure for the water-washing detoxification was operated as follows. First, different amounts of tap water were added to the pretreated CS and stirred for 1 h at 25°C. Then squeezed liquid out of the slurry until the solid content rose to 50% (wt/wt, dry base) by a hydraulic press machine at 15 MPa (P-204; Dazhang Filter Equipment, Shanghai, China) for SSF use.

### Cellulose/hemicellulose measurement and yield calculation

The contents of cellulose and hemicellulose were determined according to a two-step H_2_SO_4 _hydrolysis method put forth by the National Renewable Energy Laboratory (NREL) [14], with minor modifications. A quantity of 100 mg of thoroughly washed and dried CS were added with 1 ml of 72% (wt/wt) H_2_SO_4_, and the mixture was stirred using a glass rod until the sample was completely mixed with the acid solution. After incubation at 30°C for 1 h, the mixture was diluted by adding 28 ml of deionized water, and the diluted mixture was hydrolyzed at 121°C for 1 h. The mixture was neutralized with CaCO_3 _powder and then centrifuged at 10,000 rpm for 5 min, and the supernatant was used for HPLC analysis.

The glucose yields were calculated according to NREL LAP-009 [15] as follows:

Glucose yield = ([glucose] + 1.053 × [cellobiose])/(1.111 × [fraction] × [biomass]) × 100%,

where [glucose] is the glucose concentration in the broth after enzymatic hydrolysis, [cellobiose] is the cellobiose concentration in the broth after enzymatic hydrolysis, [biomass] is the dry biomass weight concentration at the beginning of the enzymatic hydrolysis, [fraction] is the cellulose fraction of the dry biomass (g/g), 1.053 is the conversion factor for cellobiose to equivalent glucose and 1.111 is the conversion factor for cellulose to equivalent glucose. The ethanol yields were calculated using the method described by Zhang *et al*. [13].

### Analysis of sugars, ethanol and inhibitors using HPLC

Glucose, ethanol and toxins such as furfural, HMF, acetic acid, formic acid and levulinic acid were analyzed using HPLC (LC-20AD, refractive index detector RID-10A; Shimadzu, Kyoto, Japan) with a Bio-Rad Aminex HPX-87 H column (Bio-Rad, Hercules, CA, USA) at the column temperature of 65°C. The mobile phase was 5 mM H_2_SO_4 _at the rate of 0.6 mL/min. All samples were centrifuged at 10,000 rpm for 5 min and then filtered through a 0.22-μm filter before analysis.

## Results

### Screening and identification of the detoxification strains

In the isolation step, 14 colonies with different phenotypes were isolated from the air-exposed, pretreated CS samples and were named ZN1 to ZN14, respectively. Then all 14 strains were sent for further screening.

**Figure 1:**
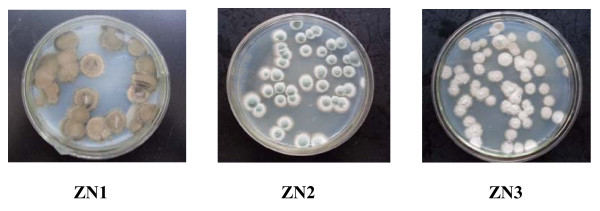
**Morphology of the isolates with toxin degradation property**. Culture conditions: 25°C for 5 days in the static incubator.

In the initial screening step, the total of 14 strains was streaked onto the screening medium to select the toxin-tolerant strains. Since acetic acid, HMF and furfural were three major products during lignocellulose pretreatment and the most toxic inhibitors to microbial growth [3,16], microbial growth behaviors on the screening medium could be used as an index of toxin tolerance. Table 1 shows that the growth performance of the strains ZN1, ZN2 and ZN3 were significantly better than the other 11 strains. Thus these three strains were selected as the candidates in the next round of detoxification screening. The morphology of the colonies ZN1, ZN2 and ZN3 on the PDA plates are shown in Figure 1.

**Table 1:** Growth behaviors of the isolated 14 strains on the PDA medium and the screening medium^a^

Isolates	Growth on PDA medium	Growth on screening medium
ZN1	+++	+++
ZN2	+++	++
ZN3	+++	+++
ZN4	+++	+
ZN5	+++	-
ZN6	+++	-
ZN7	++	-
ZN8	++	+
ZN9	+++	-
ZN10	++	-
ZN11	+++	-
ZN12	+++	+
ZN13	+++	+
ZN14	++	-

^a^- indicates no mycelium growth on the corn stover (CS); + indicates the fungal mycelium was observable but minimal; ++ indicates the fungal mycelium grew quickly and covered the surface of the solid medium of the flask; +++ indicates the fungal mycelium grew vigorously and filled the flask. The potato-dextrose-agar (PDA) plates with inocula suspension were cultured at 25°C for 5 days in the static incubator.

In the detoxification screening step, the spore suspensions of the selected strains ZN1, ZN2 and ZN3 were directly inoculated onto the steam explosion-pretreated CS samples for 5 days in solid-state culture. The detoxified CS samples were enzymatically hydrolyzed at 50°C for 12 h, and then the hydrolysate was analyzed to check the toxin degradation and the glucose release. The pretreated CS without inoculation of the selected strains was used as the control. Table 2 shows that ZN1 degraded all three toxins most efficiently compared to ZN2 and ZN3. The glucose yield after 12 h of hydrolysis was increased by 53.7% using ZN1, while the glucose yield using ZN2 and ZN3 increased by only 28.0% and 34.6%, respectively. Another finding was that ZN1 grew faster than ZN2 and ZN3 and dominated the microbial community at the end of the detoxification culture. When ZN1, ZN2 and ZN3 were mixed equivalently and inoculated on the pretreated CS material, only ZN1 survived after 1-day culture, while ZN2 and ZN3 diminished. Therefore, the strain ZN1 was chosen as the most favorable candidate biodetoxification strain, both for its best toxin degradation capacity and for growth behaviors.

**Table 2:** Glucose yield and toxins derived from the pretreated CS detoxified by the three isolates^a^

Fungal strains	Acetic acid (g/L)	Levulinic acid (g/L)	Furfural (g/L)	HMF (g/L)	Glucose yield (%)
Control	2.5 ± 0.2	3.0 ± 0.3	0.6 ± 0.1	0.9 ± 0.0	21.4 ± 0.8
ZN1	0.6 ± 0.1	0.6 ± 0.1	0	0	32.9 ± 1.2
ZN2	1.8 ± 0.2	1.8 ± 0.1	0.6 ± 0.1	0.9 ± 0.0	27.6 ± 1.3
ZN3	1.4 ± 0.1	1.4 ± 0.1	0.1 ± 0.0	0.4 ± 0.0	28.8 ± 1.0

^a^Biodetoxification was carried out in flasks at 25°C for 4 days in the static incubator. Enzymatic hydrolysis: 10% (wt/wt) solid loading, 15.0 FPU/g dry solid matter (DM) enzyme dosage, 50°C, 150 rpm in the water bath shaking incubator for 12 h. HMF, 5-hydromethylfurfural. FPU, unit of filter paper cellulase.

The ITS sequences of the three strains are shown in Table 3. The result shows that ZN1 had 98% similarity to *Amorphotheca resinae *AY251067.1 (also known as *Hormoconis resinae *and *Cladosporium resinae*) and *Cladosporium breviramosum *AF393684.2. *A. resinae *was a fungal strain known as "kerosene fungus" with a unique property of degrading petroleum and kerosene [17]. ZN2 had 98% similarity to *Penicillium polonicum *EU128628.1 [18], while ZN3 demonstrated 99% similarity to *Eupenicillium baarnense *AY213679 [19]. After comparing the colonies' morphology with that of related species, constructing the phylogenetic trees and analyzing the sequence distances among the most related isolates, the three strains were designated as *Amorphotheca resinae *ZN1, *Penicillium polonicum *ZN2 and *Eupenicillium baarnense *ZN3, respectively.

**Table 3:** ITS sequences of the three isolates^a^

*Amorphotheca resinae *ZN1	GGCTCGGAGTCTGCCTTACGGGTAGATCTCCCACCCTGTGCCATCGTTACCTTTGTTGCTTTGGCGGGCCGCCTTCGGCCGCCGGCTCACGCTGGCGCGCGCCCGCCAGAGGACCTCAACTCTTGTTTTTTAGTGTCGTCTGAGTACTATACAATCGTTAAAACTTTCAACAACGGATCTCTTGGTTCTGGCATCGATGAAGAACGCAGCGAAATGCGATAAGTAATGCGAATTGCAGAATTCAGTGAGTCATCGAATCTTTGAACGCACATTGCGCCCTGTGGTATTCCGCAGGGCATGCCTGTTCGAGCGTCATTTCAACCCTCAAGCTCTGCTTGGTGTTGGGCCCTGCCCGTCGCGGCCGGCCCTAAAATCAGTGGCGGTGCCGCTGGGCTCTGAGCGTAGTACATCTCTCGCTCCAGCGCCCCGCGGTGGCTTGCCAGAACCCCAACTTCTGTGGTTGACCTCGGATCAGGTAGGGATACCCGCTGAACTTAAGCATATCTAA
*Penicillium polonicum *ZN2	ACGAGCGAGGGGCTTTGGGTCCACCTCCCACCCGTGTTTATTTTACCTTGTTGCTTCGGCGGGCCCGCCTTTACTGGCCGCCGGGGGGCTCACGCCCCCGGGCCCGCGCCCGCCGAAGACACCCCCGAACTCTGTCTGAAGATTGAAGTCTGAGTGAAAATATAAATTATTTAAAACTTTCAACAACGGATCTCTTGGTTCCGGCATCGATGAAGAACGCAGCGAAATGCGATACGTAATGTGAATTGCAAATTCAGTGAATCATCGAGTCTTTGAACGCACATTGCGCCCCCTGGTATTCCGGGGGGCATGCCTGTCCGAGCGTCATTGCTGCCCTCAAGCCCGGCTTGTGTGTTGGGCCCCGTCCTCCGATTCCGGGGGACGGGCCCGAAAGGCAGCGGCGGCACCGCGTCCGGTCCTCGAGCGTATGGGGCTTTGTCACCCGCTCTGTAGGCCCGGCCGGCGCTTGCCGATCAACCCAAATTTTTATCCAGGTTGACCTCGGATCAGGTAGGGATACCCGCTGAACTTAAGCATATCTAAGGCGGAGGAATTA
*Eupenicillium baarnense *ZN3	CATTCACTGAGGCCTCTGGGTCCACCTCCCACCCGTGTTTATTGTACCTTGTTGCTTCGGCGGGCCCGCCTTTATGGCCGCCGGGGGGCTCACGCCCCCGGGCCCGCGCCCGCCGAAGACACCTCGAACTCTGTCTGAAGATTGTAGTCTGAGTGAAAATATAAATTATTTAAAACTTTCAACAACGGATCTCTTGGTTCCGGCATCGATGAAGAACGCAGCGAAATGCGATACGTAATGTGAATTGCAGAATTCAGTGAATCATCGAGTCTTTGAACGCACATTGCGCCCCCTGGTATTCCGGGGGGCATGCCTGTCCGAGCGTCATTGCTGCCCTCAAGCACGGCTTGTGTGTTGGGCCCCGTCCTCCGATTCCGGGGGACGGGCCCGAAAGGCAGCGGCGGCACCGCGTCCGGTCCTCGAGCGTATGGGGCTTTGTCACCCGCTCTGTAGGCCCGGCCGGCGCTTGCCGATCAACCCAAATTTTTATCCAGGTTGACCTCGGATCAGGTAGGGATACCCGCTGAACTTAAGCATATCTAAGGCGAAGA

^a^ITS, internal transcribed spacer.

### Phenotype characterization of *A. resinae *ZN1 on substrate utilization

Two control experiments were carried out to characterize the growth behaviors of *A. resinae *ZN1. The first control experiment was the effect of *A. resinae *ZN1 on the cellulose content of the CS materials. The cellulose contents of the fresh pretreated CS and the detoxified CS after 4 days of solid-state culture of *A. resinae *ZN1 were determined to be 33.4 ± 1.4% (wt/wt) and 32.6 ± 1.1% (wt/wt), respectively, indicating that the cellulose during the detoxification by *A. resinae *ZN1 was almost untouched. The reason might be that no cellulase components were secreted out the cells of *A. resinae *ZN1 to degrade cellulose in the pretreated CS. The second control experiment was the growth of *A. resinae *ZN1 on the thoroughly washed, pretreated CS materials. The result shows that no mycelia formation was observed even 9 days after *A. resinae *ZN1 was inoculated. Therefore, the toxin degradation and growth assay experiments could be carried out with the thoroughly washed pretreated CS as the substrate carrier by simply observing the mycelia formation without considering the cellulose degradation.

Table 4 shows the utilization behaviors of different lignocelluloses-derived sugars as the sole carbon sources by *A. resinae *ZN1. The result shows that *A. resinae *ZN1 could utilize all major lignocelluloses-derived sugars as the sole carbon sources, including glucose, xylose, mannose, arabinose and galactose. Particularly, *A. resinae *ZN1 utilized the two most abundant lignocelluloses-derived sugars, glucose and xylose, as the sole carbon sources quickly, while the capacity of utilizing arabinose, mannose and galactose were relatively weak.

**Table 4:** Growth behaviors of *Amorphotheca resinae *ZN1 using lignocellulose derived sugars as the sole carbon sources^a^

Time (day)	Glucose	Xylose	Mannose	Arabinose	Galactose
1	-	-	-	-	-
2	+	+	-	-	-
3	++	+	+	+	-
4	++	++	+	+	+
5	+++	+++	++	++	+
7	+++	+++	+++	++	+
9	+++	+++	+++	++	+

^a^All the sugars were set at 160 mg/g DM.-indicates no mycelium growth on the CS; + indicates fungal mycelium was observable but minimal; ++ indicates fungal mycelium grew quickly and covered the surface of the solids medium of the flask. The thoroughly washed dilute acid-pretreated CS with fungal inocula in the flasks was cultured at 25°C for 9 days in the static incubator.

Tables 5, 6, 7 and 8 show the capacity of toxin utilization by *A. resinae *ZN1 as the sole carbon sources on the thoroughly washed, pretreated CS. Table 5 shows that *A. resinae *ZN1 grew well by utilizing the two major growth inhibitors, furfural from xylose degradation and HMF from glucose degradation, as the sole carbon sources. Table 6 shows that *A. resinae *ZN1 utilized well the major organic acids derived from lignocellulose as the sole carbon sources, including acetic acid, formic acid, ferulic acid and salicylic acid. Table 7 shows that *A. resinae *ZN1 utilized well the major lignin derivatives as the sole carbon sources in a wide concentration range, such as vanillin, sesamol, 4-hydroxylbenzaldehyde, guaiacol and phenol. The concentrations of the toxins used in Tables 5, 6 and 7 already well exceeded the concentration range in the fresh pretreated CS materials. For furfural, the concentration range was limited to less than 20 mg/g DM, indicating that furfural might be the most toxic toxin for *A. resinae *ZN1 growth.

**Table 5:** Growth behaviors of *Amorphotheca resinae *ZN1 using furan-derivative toxins as sole carbon sources^a^

Time (days)	Furfural			HMF		
		
	3.9	11.6	19.3	16.7	33.3	66.7
1	-	-	-	-	-	-
2	+	-	-	-	-	-
3	+	+	-	+	-	-
4	++	++	-	+	+	-
5	+++	++	+	++	+	+
7	+++	+++	+	+++	++	+
9	+++	+++	++	+++	++	+

^a^Substrate loading units are mg/g DM.-indicates no mycelium growth on the CS; + indicates fungal mycelium was observable but minimal; ++ indicates the fungal mycelium grew quickly and covered the surface of solids medium of the flask. Thoroughly washed dilute acid-pretreated CS with fungal inocula in the flasks were cultured at 25°C for 9 days in the static incubator.

**Table 6:** Growth behaviors of *Amorphotheca resinae *ZN1 using organic acid toxins as the sole carbon sources^a^

Time (days)	Acetate acid			Formic acid			Ferulic acid			Salicylic acid	
				
	34.7	52.0	69.3	36.3	72.7	109.0	5.0	20.0	40.0	1.7	3.3
1	-	-	-	-	-	-	-	-	-	-	-
2	-	-	-	-	-	-	-	-	-	+	-
3	-	-	-	-	-	-	+	-	-	+	+
4	-	-	-	-	-	-	+	+	-	+	+
5	+	-	-	+	-	-	+	+	-	++	+
7	++	+	+	++	+	-	+	+	+	+++	++
9	+++	++	+	+++	+	-	++	+	+	+++	++

^a^Substrate loading units are mg/g DM.- indicates no mycelium growth on the CS; + indicates the fungal mycelium was observable but minimal; ++ indicates the fungal mycelium grew quickly and covered the surface of the solids medium of the flask. Thoroughly washed dilute acid-pretreated CS with fungal inocula in the flasks was cultured at 25°C for 9 days in the static incubator.

**Table 7:** Growth behaviors of *Amorphotheca resinae *ZN1 using lignin derivative toxins as the sole carbon sources^a^

Time (days)	Vanillin			Sesamol		4-hydroxybenzaldehyde		Guaiacol		Phenol	
					
	1.7	3.3	5.0	1.7	3.3	1.7	3.3	1.9	3.7	1.7	3.3
1	-	-	-	-	-	-	-	-	-	-	-
2	-	-	-	+	-	+	-	+	+	+	-
3	+	+	+	+	+	+	+	+	+	+	+
4	++	+	+	+	+	+	+	+	+	+	+
5	++	++	+	++	+	++	+	++	+	++	+
7	+++	++	+	+++	+++	+++	++	+++	+++	+++	++
9	+++	++	+	+++	+++	+++	++	+++	+++	+++	++

^a^Substrate loading unit: mg/g DM.-indicates no mycelium growth on the CS; + indicates the fungal mycelium was observable but minimal; ++ indicates the fungal mycelium grew quickly and covered the surface of the solids medium of the flask. Thoroughly washed dilute acid-pretreated CS with fungal inocula in the flasks was cultured at 25°C for 9 days in the static incubator.

**Table 8:** Growth behaviors of *Amorphotheca resinae *ZN1 using hydrocarbons as the sole carbon sources^a^

Time (days)	Kerosene	Dodecane (C12 alkane)		
		
	267	247	494	741
1	-	-	-	-
2	-	-	-	-
3	-	-	-	-
4	-	-	-	-
5	-	-	-	-
7	+	+	+	-
9	++	+	+	+

^a^Substrate loading unit: mg/g DM.-indicates no mycelium growth on the CS; + indicates the fungal mycelium was observable but minimal; ++ indicates the fungal mycelium grew quickly and covered the surface of the solids medium of the flask. Thoroughly washed dilute acid-pretreated CS with fungal inocula in the flasks was cultured at 25°C for 9 days in the static incubator.

Table 8 shows that *A. resinae *ZN1 utilized well the two hydrocarbon compounds, kerosene and dodecane (a typical C12 alkane), as the sole carbon sources. The result confirmed the unique and intrinsic property of *A. resinae *species on hydrocarbon metabolism and provided the biological evidence of the strain identification, besides the 18 S rDNA blasting result [17].

The above results indicate that the use of solid-state culture on the pretreated CS solids by *A. resinae *ZN1 could be a reasonable option instead of submerged liquid fermentation in the hydrolysate after hydrolysis of the pretreated CS. The reasons include at least that (1) higher toxin concentrations in the pretreated CS solids accelerated toxin degradation compared to diluted toxin concentrations in the hydrolysate slurry after the hydrolysis of the pretreated CS, (2) the sugar loss in the pretreated CS solids was reduced because of the very low concentrations of the free monosaccharide sugars (especially glucose) in the pretreated CS solids compared to that in the CS hydrolysate, (3) there was no observable degradation of cellulose by the solid-state culture of *A. resinae *ZN1 and (4) the toxin inhibition was alleviated to the cellulase in subsequent enzymatic hydrolysis of the pretreated CS after solid-state biodetoxification.

### Toxins degradation performance of *A. resinae *ZN1 on pretreated CS

**Figure 2:**
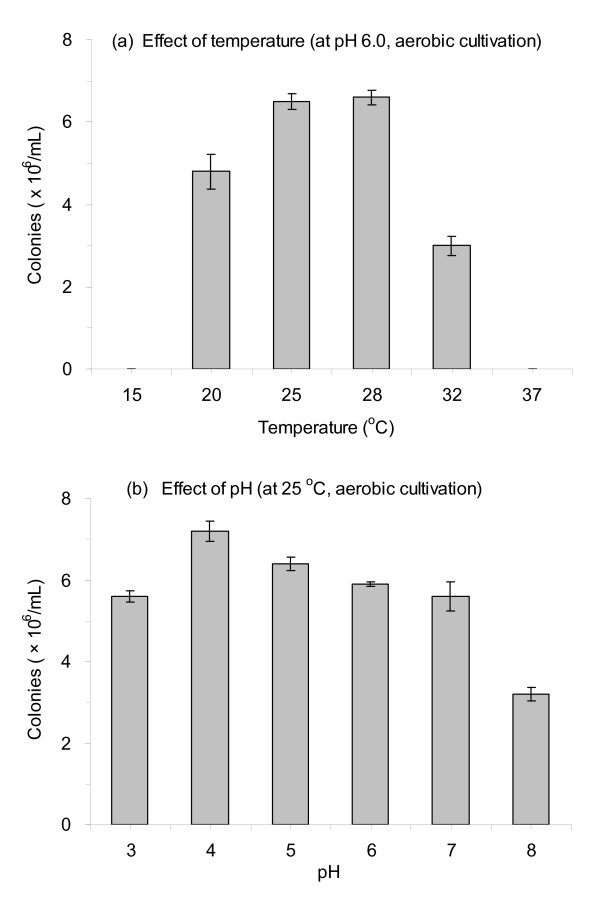
**Effect of culture conditions on the growth of *Amorphotheca resinae *ZN1**. **(a) **Effect of temperature. **(b) **pH. Growth conditions on pretreated corn stover (CS) material: The solid content of the pretreated CS was 40% (wt/wt), and the culture lasted for 3 days in the static incubator. Culture conditions on potato-dextrose-agar (PDA) plates: 25°C for 4 days in the static incubator.

Figure 2 shows the growth behaviors of *A. resinae *ZN1 on the pretreated CS by measuring the colony numbers formed during the solid-state culture. Figure 2a shows that the growth temperature range of *A. resinae *was between 20°C and 32°C, with the optimal range of 25-28°C, similar to most of the fungal strains. Figure 2b shows that *A. resinae *grew well in a wide pH range with an optimum of pH 4-6. The growth behaviors with different oxygen levels (data not shown) indicated that *A. resinae *ZN1 grew well in both aeration conditions, and, perhaps, the aerobic condition preferred its growth slightly.

**Figure 3:**
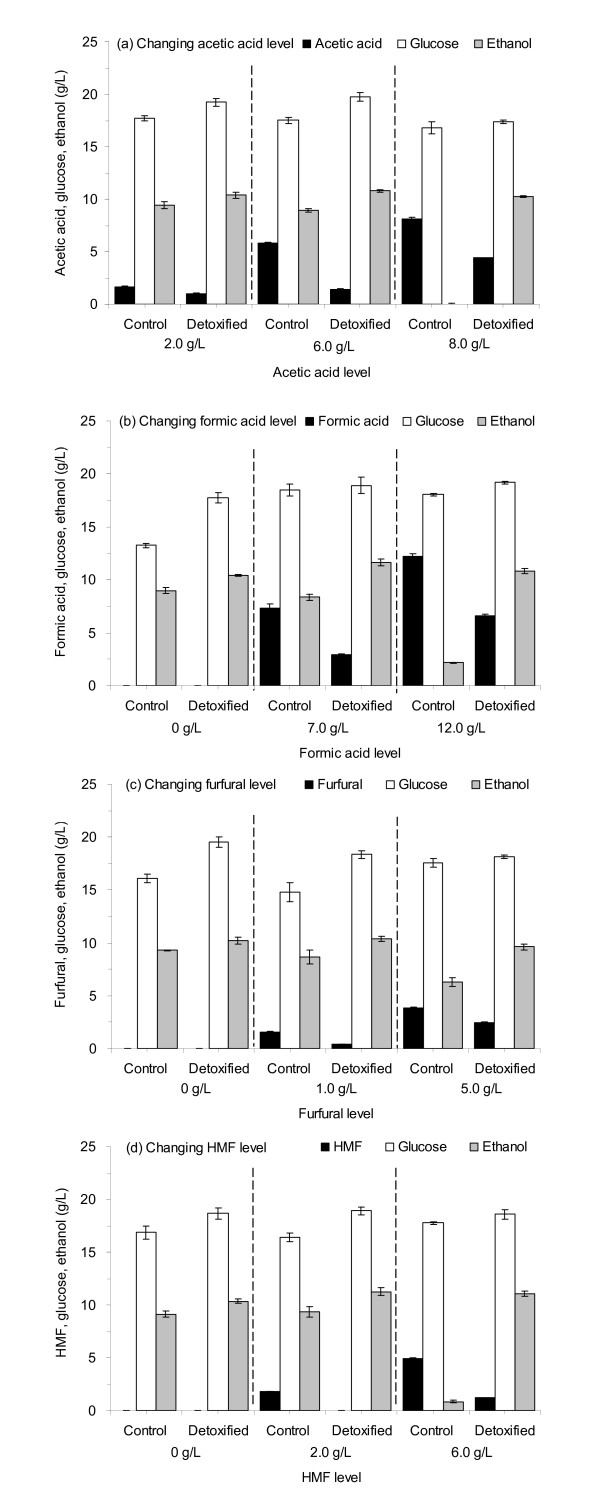
**Degradation of single toxin with changing concentrations on the pretreated CS by *A***. *resinae *ZN1 and the effect on the consequent simultaneous saccharification and ethanol fermentation (SSF). **(a) **Changing acetic acid level. **(b) **Changing formic acid level. **(c) **Changing furfural level. **(d) **Changing 5-hydromethylfurfural (HMF) level. Degradation conditions were 5 days at 25°C in the static incubator. SSF conditions were 10% (wt/wt) of the solid loading, 15.0 FPU/g dry solid matter (DM), at pH 5.0, 50°C at the first 12-h prehydrolysis stage and 37°C at the sequential SSF stage, 150 rpm, 50 ml/250 ml flask, incubated in the water bath shaking incubator. FPU, unit of filter paper cellulase.

Figure 3 shows that the degradation performance of different single toxin supplemented onto the pretreated CS solids by *A. resinae *ZN1. The detoxified CS was then used for solid-state culture and SSF in flasks. Figure 3a shows that at different acetic acid levels (2.0 g/L, 6.0 g/L and 8.0 g/L), the acetic acid concentrations in the detoxified CS fermentation broth significantly decreased compared to that found using nondetoxified CS. The glucose and ethanol yields also increased compared to the control. When the acetic acid level reached 8.0 g/L, the detoxification by *A. resinae *ZN1 became the inevitable step for ethanol fermentation: no ethanol formed in the control flask. Similarly, Figures 3b, 3c and 3d show that the formic acid, furfural and HMF decreased, respectively, after the solid-state detoxification of *A. resinae *ZN1 on the pretreated CS and concomitantly the increase of glucose and ethanol yields. At higher levels of the toxins, the biodetoxification of *A. resinae *ZN1 showed its unique function for accelerating the SSF process rate.

**Figure 4:**
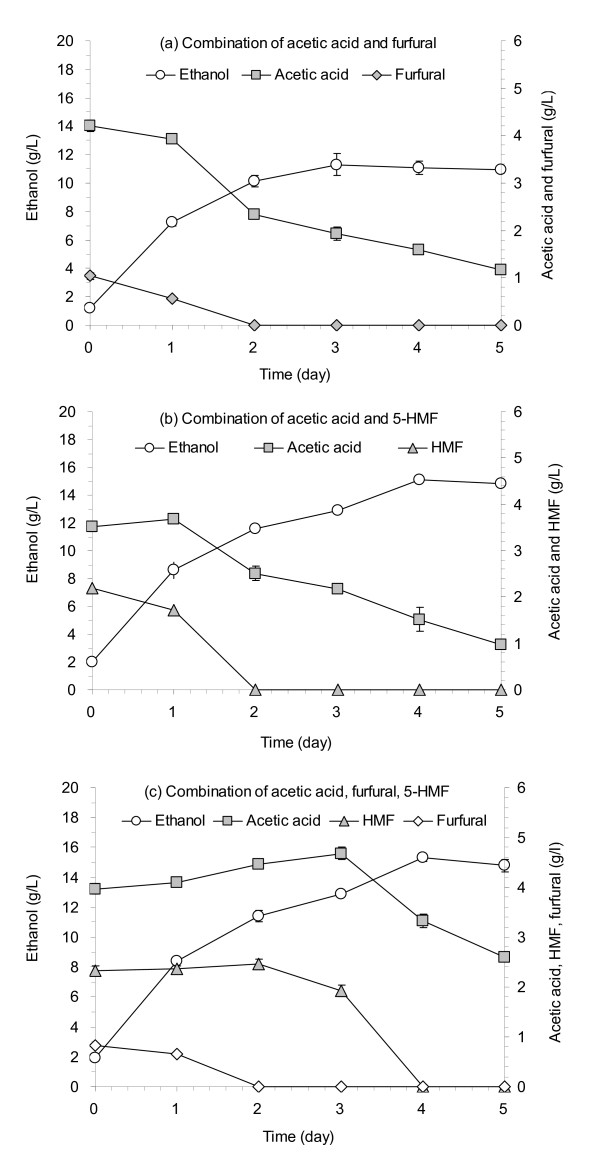
**Degradation of complex toxins on the pretreated CS by *A***. *resinae ZN1 *and the effect on the consequent SSF. **(a) **Acetic acid and furfural. **(b) **Acetic acid and HMF. **(c) **Acetic acid, furfural, and HMF. Degradation conditions: 5 days at 25°C in the static incubator. SSF conditions: 10% (wt/wt) of the solids (DM) loading, 15.0 FPU/g DM, at pH 5.0, 50°C at the first 12-h prehydrolysis stage and 37°C at the sequential SSF stage, 150 rpm, 50 ml/250 ml flask, incubated in the water bath shaking incubator. FPU, unit of filter paper cellulase.

Figure 4 shows the degradation of the mixed toxins supplemented onto the pretreated CS solids by *A. resinae *ZN1. Figures 4a and 4b show the binary combination of acetic acid and furfural and of acetic acid and 5-HMF. Figure 4c shows the tertiary combination of acetic acid, furfural and 5-HMF. The results show that furfural was completely degraded prior to acetic acid (Figure 4a) followed by HMF (Figure 4b). Figure 4c indicates that furfural degradation started first, HMF started to be degraded after furfural was almost completely degraded and acetic acid started to be degraded approximately after both furfural and HMF were completely degraded. The ethanol titer increased with decreasing toxin concentrations in all three cases.

### SSF under high solid loading of biodetoxified CS

**Figure 5:**
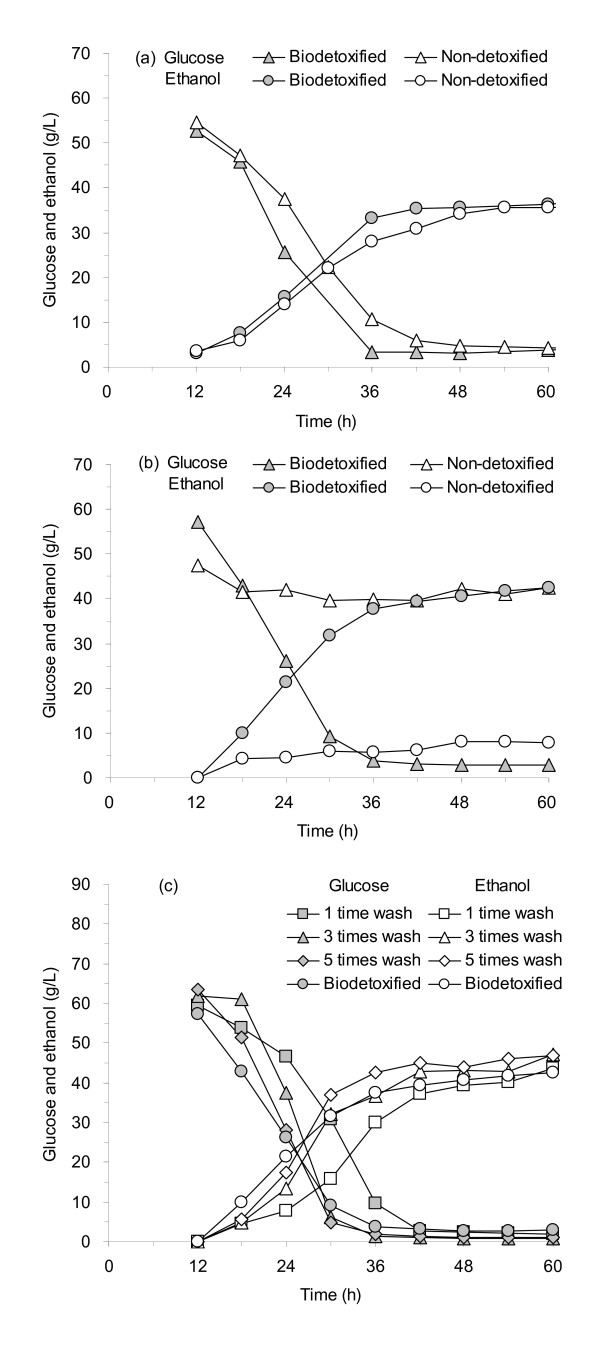
**SSF of different pretreated CS materials**. **(a) **Steam explosion-pretreated CS after 4 days of biodetoxification. **(b) **Dilute sulfuric acid-pretreated CS after 4 days of biodetoxification. **(c) **Dilute sulfuric acid-pretreated CS after water-washing detoxification at different liquid-to-solids ratios. Biodetoxification conditions for different pretreated CS: 4 days at 25°C in the static incubator. SSF conditions: 30% (wt/wt) solids loading, 15.0 FPU/g DM, pH 5.0, in the helical stirring bioreactor at 150 rpm. FPU, unit of filter paper cellulase.

Figure 5 shows the SSF of the different pretreated CS after biodetoxification by *A. resinae *ZN1 at solid loading of 30% (wt/wt, dry base). Two feedstocks, the steam explosion-pretreated and the dilute sulfuric acid-pretreated CS material, were detoxified using the solid-state culture of *A. resinae *ZN1 for 4 days and then fed into the helical stirring bioreactor for SSF. Figure 5a shows that the glucose consumption and the ethanol production rates using the steam explosion-pretreated CS after biodetoxification were greater than that using the fresh pretreated CS within the first 48 h, although the final ethanol concentrations reached the same level after 60-h fermentation. The results indicated that the toxin removal by biodetoxification lessened the toxin inhibition to the fermenting yeast to a large extent and thus enhanced ethanol productivity. Figure 5b shows the SSF using the dilute sulfuric acid CS after biodetoxification was finished within 36 or 48 h, while the normal fermentation using the fresh dilute sulfuric acid-pretreated CS did not occur. The result indicates that the solid-state detoxification of *A. resinae *ZN1 significantly improved the SSF performance at high solid loading and worked for different pretreated CS feedstocks. No *A. resinae *ZN1 growth was found during the 60-h SSF operation by spreading samples taken periodically on the PDA plates, indicating that *A. resinae *ZN1 did not survive in the anaerobic ethanol fermentation.

Figure 5c and Table 9 show comparisons between the biodetoxification of *A. resinae *ZN1 and water washing detoxification methods. Figure 5c shows that glucose utilization and ethanol production using the biodetoxified CS were similar to that using water washing detoxification. The water washing method removed most of the toxins; however, the water washing method resulted in considerable loss of solids in addition to generating massive sulfuric acid containing wastewater (Table 9). If the solids loss in the water washing is considered, the overall ethanol yield using the biodetoxified CS was approximately 8% greater than the best result using the water washing CS.

**Table 9:** Comparisons of the two detoxification methods for pretreated CS^a^

	Glucose	Xylose	Acetic acid	Levulinic acid	HMF	Furfural	DM loss	Ethanol yield	Water usage
	(mg/g DM)						(%)	(%, wt/wt)	(kg/kg DM)
Original	10.7 ± 0.2	52.6 ± 2.1	12.3 ± 0.1	2.2 ± 0.2	4.1 ± 0.3	6.2 ± 0.5	0.0	10.3	0.0
Biodetoxified	4.0 ± 0.4	22.1 ± 0.8	0.1 ± 0.1	0.6 ± 0.2	3.4 ± 0.2	0.0 ± 0.1	0.0	56.5	0.0
water washing									
L/S ratio 1:1	3.1 ± 0.2	15.3 ± 1.3	3.3 ± 0.0	0.6 ± 0.2	0.8 ± 0.2	1.1 ± 0.1	18.5 ± 0.8	47.4	2.0 ± 0.3
L/S ratio 3:1	2.0 ± 0.0	7.6 ± 0.5	2.1 ± 0.2	0.5 ± 0.3	0.7 ± 0.1	0.9 ± 0.2	21.3 ± 0.6	49.3	6.0 ± 0.6
L/S ratio 5:1	1.2 ± 0.1	6.1 ± 0.0	1.4 ± 0.3	0.2 ± 0.1	0.4 ± 0.1	0.8 ± 0.2	27.2 ± 1.2	45.9	10.0 ± 1.0

^a^L/S ratio indicates the freshwater usage vs. the weight of the pretreated CS (wt/wt) during the washing step. Conditions for biodetoxification: 25°C for 4 days in a static incubator. Conditions for SSF: 30% (wt/wt) solid loading, 15.0 FPU/g DM, pH 5.0, in the helical stirring bioreactor at 150 rpm. FPU, unit of filter paper cellulase.

### Application of biodetoxification method to different lignocellulose feedstocks

**Figure 6:**
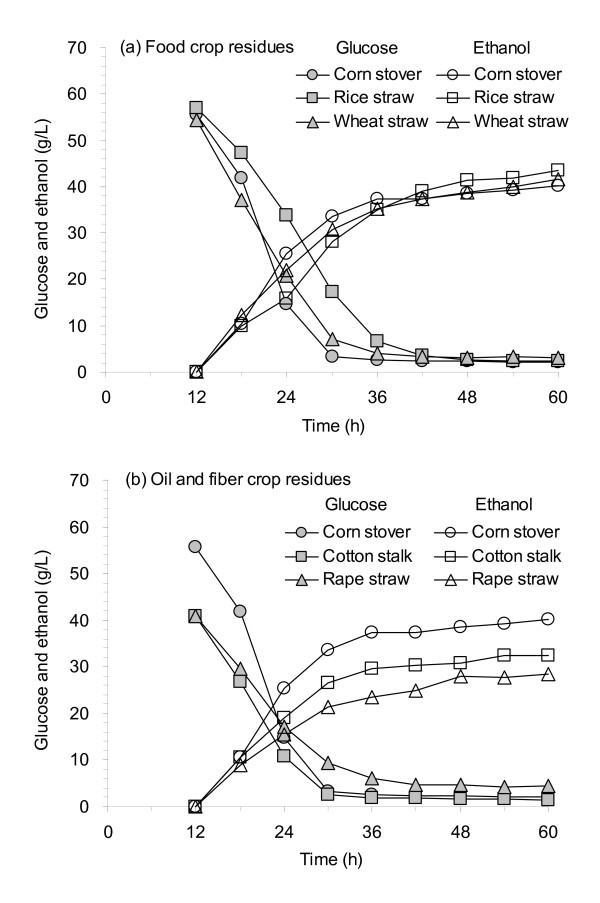
**Application of the biodetoxification method to various lignocellulose feedstocks**. **(a) **Food crop residues. **(b) **Oil and fiber crop residues. Biodetoxification conditions for various lignocellulose feedstocks: 3 days at 25°C in the static incubator. SSF conditions: 30% (wt/wt) solids loading, 15.0 FPU/g DM, pH 5.0, in the helical stirring bioreactor at 150 rpm. FPU, unit of filter paper cellulase.

Figure 6 shows the SSF performance of the biodetoxification by *A. resinae *ZN1 on pretreated CS was tested on different lignocellulose materials. Five lignocellulose materials were collected, including three food crop residues, CS, wheat straw and rice straw, as well as two oil and fiber crop residues, cotton stalk and rape straw. The same mechanical milling, the dilute sulfuric acid pretreatment, the solid-state detoxification by *A. resinae *ZN1 and the high solid loading SSF procedure were followed for all five feedstocks. No cell growth or ethanol formation was detected in the SSF operation using the fresh pretreated materials of all five lignocellulose feedstocks without biodetoxification, similar to the result in Figure 5b with the nonbiodetoxified CS.

Figure 6a shows that the SSF performance for the three food crop residues was similar, although the glucose utilization and the ethanol production rate showed some minor differences, with the order of CS > wheat straw > rice straw. Figure 6b shows that the performance of the two oil and fiber crop residues was relatively poorer compared to that of the food crop residues with the lowered ethanol yield up to 20-30%, but still worked for these residues. Perhaps the chemicals and inherent structure of the food crop residues might be different from the oil and fiber crop residues, and the pretreatment and biodetoxification procedures for the nonfood crop residues needed to be optimized on an individual basis, while for food crop residues the procedures might be universal from pretreatment to biodetoxification and to SSF operation.

## Discussion

Ethanol fermentation strains as well as cellulase enzymes were severely inhibited by the toxic compounds generated in the harsh pretreatment of lignocellulose. Thus the efficient removal of the toxins is a requisite step for the biological conversion using lignocellulose feedstock. To avoid the drawbacks in routine detoxification, biodetoxification now is being considered as a promising method for toxin removal at mild conditions, low energy input and zero wastewater release. Currently, many bacteria and fungi were isolated and identified to have toxin degradation capacity, but the ethanol fermentation practice using biodetoxified lignocellulose feedstocks has not been demonstrated.

In this study, the newly isolated *A. resinae *ZN1 from the pretreated CS sample showed many advantages. *A. resinae *ZN1 grew particularly fast and dominated the microbial community during the detoxification culture on the intensively pretreated lignocellulose material with high concentration of inhibitors. This property lessened the possibility of contamination by other microbial organisms because of its fast growth advantage. *A. resinae *ZN1 was able to utilize all the known toxin inhibitors as the sole carbon sources for its growth and showed a wide applicability to detoxification of various lignocellulose feedstocks. Also, no cellulose degradation was observed during the *A. resinae *ZN1 culture on solid CS, and thus the cellulose loss could be avoided in the biodetoxification step. On the other hand, *A. resinae *ZN1 diminishes spontaneously in the anaerobic fermentation.

The solid-state culture model was applied for biodetoxification by *A. resinae *ZN1 on the solid pretreated materials instead of performing biodetoxification in the liquid hydrolysate. The solid-state culture fits the fungus habitat with relatively sufficient oxygen supply compared to the liquid fermentation in the hydrolysate. The conditions were also preferable for quick toxin degradation: high toxin concentration, low sugar contents (xylose was relatively high, but glucose formed was negligible), no energy requirement and no wastewater generation. In other words, the target inhibitors were in high concentrations and the unwanted sugars were low. This was particularly important because this strain (and perhaps most other strains) took up glucose better than inhibitor substances, although it was able to take inhibitors as the sole carbon sources. Finally, the solid-state culture of biodetoxification proved to be a fast process: more than half of toxins were degraded within 1-2 days, and 3-4 days could be long enough for performing the consequent SSF process normally.

The disadvantage of the current biodetoxification by *A. resinae *ZN1 was its consumption of considerable xylose contained in the pretreated feedstocks, although it may be considered to be a partial solution for xylose utilization. Also, the *A. resinae *ZN1 biodetoxification rate was still slow compared to the best processing time, which may be limited to 1 day.

## Conclusions

In conclusion, biodetoxification by *A. resinae *ZN1 provided a fast and efficient biodetoxification method for removing toxins generated during intensive lignocellulose pretreatment, and its advantages made it possible for potential industrial application. The advantages over the known biodetoxification include zero energy input, zero wastewater generation, complete toxin degradation, processing on solid pretreated material, no need for sterilization and a wide lignocellulose feedstock spectrum. Mechanisms such as metabolites and pathways responsible for detoxification are under investigation. The genome sequencing of *A. resinae *ZN1 is on the way, and bioinformation knowledge is expected to be used for metabolism pathway elucidation and genetic modification.

## Competing interests

The authors declare that they have no competing interests.

## Authors' contributions

JZ and ZZ participated in the design of the study, performed the experimental work and wrote the draft manuscript. XFW carried out the phenotype characterization studies. NW and WW carried out the molecular identification studies. JB participated in the design of the study and wrote and commented on the manuscript. All authors took part in planning the study, checking the results and writing the manuscript. All authors read and approved the final manuscript.
